# Reimagining care of people living with rare diseases with artificial intelligence

**DOI:** 10.1371/journal.pmed.1004966

**Published:** 2026-02-26

**Authors:** Tudor Groza, Gareth Baynam, Saumya Shekhar Jamuar

**Affiliations:** 1 Bioinformatics Institute (BII), Agency for Science, Technology and Research (A*STAR), Singapore, Singapore; 2 Maternal and Child Health Research Institute, KK Women’s and Children’s Hospital, Singapore, Singapore; 3 Genetics Service, KK Women’s and Children’s Hospital, Singapore, Singapore; 4 SingHealth Duke-NUS Institute of Precision Medicine, Singapore, Singapore; 5 Duke-NUS School of Medicine, Singapore, Singapore; 6 Rare Care Centre, Perth Children’s Hospital, Perth, Western Australia, Australia; 7 SingHealth Duke-NUS Genomic Medicine Centre, Singapore, Singapore

## Abstract

In this Perspective, Tudor Groza and colleagues discuss how artificial intelligence (AI) can transform rare disease care when organized around the patient journey, outlining a patient-clinician-AI triad spanning early detection, diagnosis, clinical trials, and individualized therapies.

## Teaser

Artificial intelligence (AI) can transform rare disease care when organized around the patient journey. We outline a patient–clinician–AI triad spanning early detection, diagnosis, clinical trials, and individualized therapies.

Rare diseases collectively affect hundreds of millions of people worldwide, yet individual conditions are rare, heterogeneous, and often poorly characterized. Patients and families frequently experience years of misdiagnoses, fragmented care, and social disruption—a journey often described as the “diagnostic odyssey.” Rare diseases therefore function both as a stress test for health systems and as a proving ground for digital health infrastructures and emerging artificial intelligence (AI) technologies [1,2].

**Fig 1:**
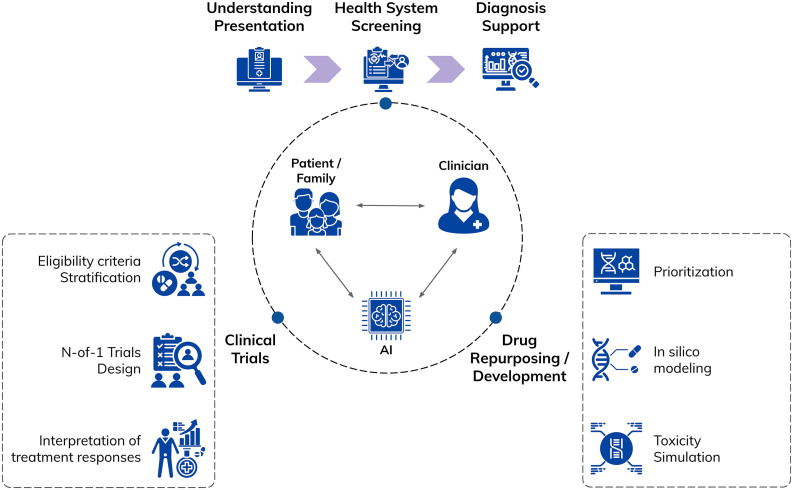
Artificial intelligence across the rare disease life course: the patient–clinician–AI triad. A conceptual view of the patient–clinician–AI triad, showing how AI supports the care pathway from initial presentation, health system screening, and diagnosis support through to clinical trial design (including eligibility, stratification, and N-of-1 trials), as well as drug repurposing/development via *in silico* modeling, toxicity simulation, and prioritization. Icons made by Freepik from https://www.flaticon.com.

Recent work has outlined how AI could support rare conditions across public health surveillance, symptom matching, and digital therapeutics, offering a system-level perspective rather than a narrow diagnostic focus. At the same time, analyses of AI in primary care and surveys of clinicians highlight persistent barriers to implementation, including workflow integration, data quality, trust, and accountability [2,3]. In this Perspective, we argue that progress in AI for rare diseases should be organized along the patient journey—from early suspicion, through diagnosis and treatment development—and grounded in a “triad” between patient-family experts, clinicians, and AI, with each contributing distinct and complementary forms of expertise (Fig 1).

## Overcoming challenges of identifying and diagnosing rare diseases

Many people with rare diseases leave extensive digital signals in electronic health records (EHRs) long before a rare condition is suspected. These signals include repeated non-specific presentations, unusual constellations of symptoms, and clusters of abnormal laboratory findings distributed across multiple encounters and care settings. In response, AI-based approaches have been developed to retrospectively and prospectively identify such patterns and flag patients who may warrant further evaluation.

Existing methods span a spectrum from relatively transparent, rule-based phenotypic scores to data-driven models that learn patterns across longitudinal records and clinical narratives. Concrete examples illustrate how these approaches translate routine EHR data into actionable rare disease signals. For example, Cohen and colleagues [4] used natural language processing to pre-screen for aromatic L-amino acid decarboxylase deficiency (AADCd) by mining unstructured clinical notes for recurring combinations of early, non-specific neurological features (e.g., hypotonia, movement disorders, developmental delay) distributed across encounters and specialties. Patients were ranked by similarity to a prototypical AADCd phenotype, and retrospective evaluation showed that confirmed or likely cases could have been flagged years earlier using documentation already present in the EHR. Similarly, Faviez and colleagues [5] applied a deep-learning natural language processing pipeline to enrich longitudinal phenotyping for Jeune syndrome, extracting and normalizing rich skeletal and extra-skeletal features and improving discrimination from phenotypically overlapping conditions. Together, these studies show how AI can integrate repeated non-specific encounters and fragmented symptom documentation into disease-specific phenotypic signatures that support earlier recognition in real-world clinical data. However, despite these promising results, the applications remain disease-specific and retrospective, and their generalizability and prospective impact require further validation.

At the health-system level, several deployments now suggest that AI-based rare disease screening is technically feasible at scale, with tools scanning hundreds of thousands of records across care networks and identifying patients for targeted review [6]. These deployments also reveal important constraints. System-wide screening requires sustained computational infrastructure, robust integration with heterogeneous clinical systems, and careful calibration of alert thresholds so that flagged patients can be reviewed without overwhelming services. Data fragmentation and variable data quality remain major barriers, as rare disease signals are often split across institutions and care episodes. Governance and trust are therefore central, particularly when screening undiagnosed populations.

Once a rare disease is suspected, AI tools can also support diagnostic reasoning by integrating genetic, phenotypic, and imaging data. AI-assisted pipelines can prioritize candidate variants, match symptom profiles to diagnoses, and synthesize multimodal evidence with performance levels that in some settings (when detailed phenotyping is available) approach that of expert clinicians [1,2]. Diagnosis, however, is rarely a single event. It unfolds over time as symptoms evolve and new information accumulates. Consistent with this, evidence suggests that the greatest clinical value arises when AI tools are embedded within broader diagnostic pathways that include specialist review, confirmatory testing, counseling, and follow-up, rather than operating as standalone decision-makers (Fig 1).

## Clinical trials for rare diseases: How can AI help?

Once a diagnosis is made, however, treatment is far from guaranteed. Despite the identification of more than 7,000 rare diseases, effective disease-modifying treatments exist for fewer than 10% of them, leaving the vast majority of patients without evidence-based therapeutic options. Accelerating the generation of high-quality clinical trial evidence is therefore a central unmet need in rare disease care [7]. The same features that complicate diagnosis—i.e., small patient numbers, clinical heterogeneity, and fragmented data—also challenge conventional clinical trial designs for rare diseases. Around 30%–50% of interventional rare disease trials enroll fewer than 50 participants, and many fail to reach planned sample sizes because patients are geographically dispersed and narrowly phenotyped [8]. As a result, trials often rely on single-arm designs with external or natural-history comparators, enriched inclusion criteria, and short, clinically meaningful endpoints rather than large randomized trials [9]. Analytical strategies also differ: Bayesian methods that incorporate prior knowledge and real-world evidence are increasingly recommended where frequentist approaches would be infeasible [8,10]. These adaptations are now established practice but introduce additional methodological and operational complexity.

Within this constrained landscape, AI is being explored as a set of targeted tools supporting multiple stages of the trial design-analysis pipeline. In drug discovery and repurposing, AI-driven *in silico* modeling and disease-drug network analyses have been used to prioritize candidate compounds when biological knowledge and patient numbers are limited [11]. In trial planning, machine learning models applied to natural-history and registry data can simulate eligibility criteria, stratification schemes, and endpoint choices, helping to maximize information yield from very small cohorts [12]. During trial conduct, quantitative systems pharmacology and AI-enhanced disease progression models can support adaptive dosing and interim decision-making, particularly in Bayesian adaptive or platform trials [10].

For ultra-rare conditions and highly individualized interventions, such as bespoke antisense oligonucleotide therapies, these approaches converge in N-of-1 trial frameworks [13]. Here, AI can assist in performing toxicity simulation (to predict off-target binding, immune activation, and sequence-specific safety liabilities), interpreting repeated on–off treatment responses, or finding common pathways to create basket trials across similar or related clinical conditions. AI-derived digital, imaging, or biomarker endpoints may further increase sensitivity to change over short time frames, enabling smaller and shorter studies [12].

Moving from retrospective analyses to prospective use requires embedding AI directly into clinical workflows so that it generates real-time, actionable predictions at the point of care. This shift will have to rely on prospective, pre-specified validation studies, silent-mode deployment before activation, predefined performance thresholds, continuous outcome tracking, and regulatory co-development that clarifies accountability. In this model, AI evolves from a post-hoc analytic layer into a continuously monitored decision-support partner whose predictions are explicitly tested against downstream patient outcomes in routine care.

## Integrating AI for rare diseases: The patient–clinician–AI “triad”

Across the rare disease life course, patients and families often become the most knowledgeable experts on their specific condition, accumulating experiential knowledge through years of observation and self-education. Building on the challenges described above, a realistic role for AI in rare diseases is not a clinician-AI partnership alone, but a three-way “triad” in which patient-family experts, clinicians, and AI systems contribute distinct and complementary forms of expertise.

In this framework, patients and families contribute lived, contextual knowledge; clinicians provide medical judgment, accountability, and care coordination; and AI integrates heterogeneous data to identify patterns and support decision-making across diagnosis, care, and research (Fig 1). Elements of this framework already exist: Patient and caregiver-reported outcomes, home monitoring data, and patient-maintained records are increasingly used in care and research, while clinicians synthesize these inputs with clinical findings. AI systems can augment this process by structuring free-text narratives, aligning patient-generated information with clinical records, and linking individual cases to wider knowledge bases—capabilities that are technically feasible today but variably deployed.

Crucially, the triad emphasizes bidirectional learning rather than one-way automation. AI systems can only generalize beyond single cases if outcomes, responses to interventions, and lived experiences are systematically fed back into shared datasets, transforming individual journeys into collective knowledge. In principle, this feedback loop could help address the data scarcity that limits screening, trial design, and treatment development, though evidence for sustained benefit across diverse settings remains limited.

Taken together, the patient–clinician–AI triad provides a coherent framework for aligning technological possibilities with the realities of rare disease care. It clarifies where AI can add value—by integrating data and amplifying learning across cases—while underscoring that responsibility, trust, and decision-making must remain shared.

## Abbreviations

AADCd: aromatic L-amino acid decarboxylase deficiency
AI: artificial intelligence
EHRs: electronic health records
